# The New York Medical and Surgical Reporter

**Published:** 1845-12

**Authors:** 


### THE NEW YORK MEDICAL AND SURGICAL REPORTER.

We have received three numbers of a journal with the above title, edited by *Clarkson T. Collins*, M.D., which is to be issued once a fortnight, in numbers of eighteen pages, at $2 per annum. As its name imports, it is to be a reporter of matters medical and surgical, and will contain the clinical lectures of the most eminent medical men in the city of New York. The numbers before us embrace several such lectures from Professors Mott and Parker. Much original matter may be expected in the pages of a journal which draws from such sources as appear to be at the command of the Reporter, and we shall be disappointed if it prove not to be a valuable repository of facts and observations. Y.

